# Extracellular signals and receptor-like kinases regulating ROP GTPases in plants

**DOI:** 10.3389/fpls.2014.00449

**Published:** 2014-09-22

**Authors:** Kaori N. Miyawaki, Zhenbiao Yang

**Affiliations:** ^1^Shanghai Center for Plant Stress BiologyShanghai, China; ^2^Center for Plant Cell Biology, Department of Botany and Plant Sciences, University of CaliforniaRiverside, CA, USA

**Keywords:** ROP GTPase, RLK, auxin, abscisic acid (ABA), calcium

## Abstract

Rho-like GTPase from plants (ROPs) function as signaling switches that control a wide variety of cellular functions and behaviors including cell morphogenesis, cell division and cell differentiation. The *Arabidopsis thaliana* genome encodes 11 ROPs that form a distinct single subfamily contrarily to animal or fungal counterparts where multiple subfamilies of Rho GTPases exist. Since Rho proteins bind to their downstream effector proteins only in their GTP-bound “active” state, the activation of ROPs by upstream factor(s) is a critical step in the regulation of ROP signaling. Therefore, it is critical to examine the input signals that lead to the activation of ROPs. Recent findings showed that the plant hormone auxin is an important signal for the activation of ROPs during pavement cell morphogenesis as well as for other developmental processes. In contrast to auxin, another plant hormone, abscisic acid, negatively regulates ROP signaling. Calcium is another emerging signal in the regulation of ROP signaling. Several lines of evidence indicate that plasma membrane localized-receptor like kinases play a critical role in the transmission of the extracellular signals to intracellular ROP signaling pathways. This review focuses on how these signals impinge upon various direct regulators of ROPs to modulate various plant processes.

## INTRODUCTION

The Rho family of small GTPases is conserved and plays pivotal roles in cellular signaling in all eukaryotic kingdoms. In animals and fungi, the Rho family is divided into Rac, Rho, and CDC42 subfamilies, each with unique functions. Plants contain a single subfamily, named Rho-like GTPase from plants (ROPs) which appears to have evolved prior to the divergence of fungal and animal Rac, Rho, and CDC42. The *Arabidopsis thaliana* genome encodes 11 ROPs and most of them are associated with the plasma membrane (PM), where they transmit the signal from membrane-localized receptors (Li et al., 2001; Fu et al., 2002; Gu et al., 2004; Nibau et al., 2006; Yang, 2008; Craddock et al., 2012). Thus the regulation of their membrane association is important for ROP signaling. ROP signaling can be negatively regulated by guanine nucleotide dissociation inhibitors (GDIs), which are responsible for the dissociation of ROPs from the PM and by sequestering them in the cytosol in inactive GDP-bound forms. When associated with the PM, ROP proteins shuttle between inactive GDP-bound form and active GTP-bound form. They bind to their downstream effector proteins only when they are in the GTP-bound active status. Once the upstream signals are perceived by receptors, guanine nucleotide exchange factors (GEFs) replace the GDP bound to ROPs with GTP. RopGEFs have a conserved plant specific ROP nucleotide exchanger (PRONE) domain for GEF activity. In contrast to RopGEFs, GTPase-activating proteins (GAPs) promote GTP hydrolysis of ROP proteins. The activation of ROPs by upstream factor(s) is a critical step in the regulation of ROP signaling. Several lines of evidence show that ROPs have roles in signaling pathway mediated by some plant hormones, such as auxin and abscisic acid (ABA). Recent findings showed that auxin is an important signal for the activation of ROPs during leaf epidermal pavement cell (PC) morphogenesis as well as in other developmental processes such as root hair development. In both cases, PM localized-receptor like kinases (RLKs) play critical roles for transmitting an extracellular auxin signal to intracellular ROP signaling. By contrast, ROPs are inactivated by ABA. In addition to plant hormones, calcium is an emerging signal in the regulation of ROP signaling in pollen tube growth. This review focuses on the mechanism underlying upstream regulation of ROP signaling and on how these signals impinge upon various direct regulators of ROPs to modulate various plant developmental processes.

### EXTRACELLULAR AUXIN SIGNAL ACTIVATES ROP SIGNALING

**FIGURE 1:**
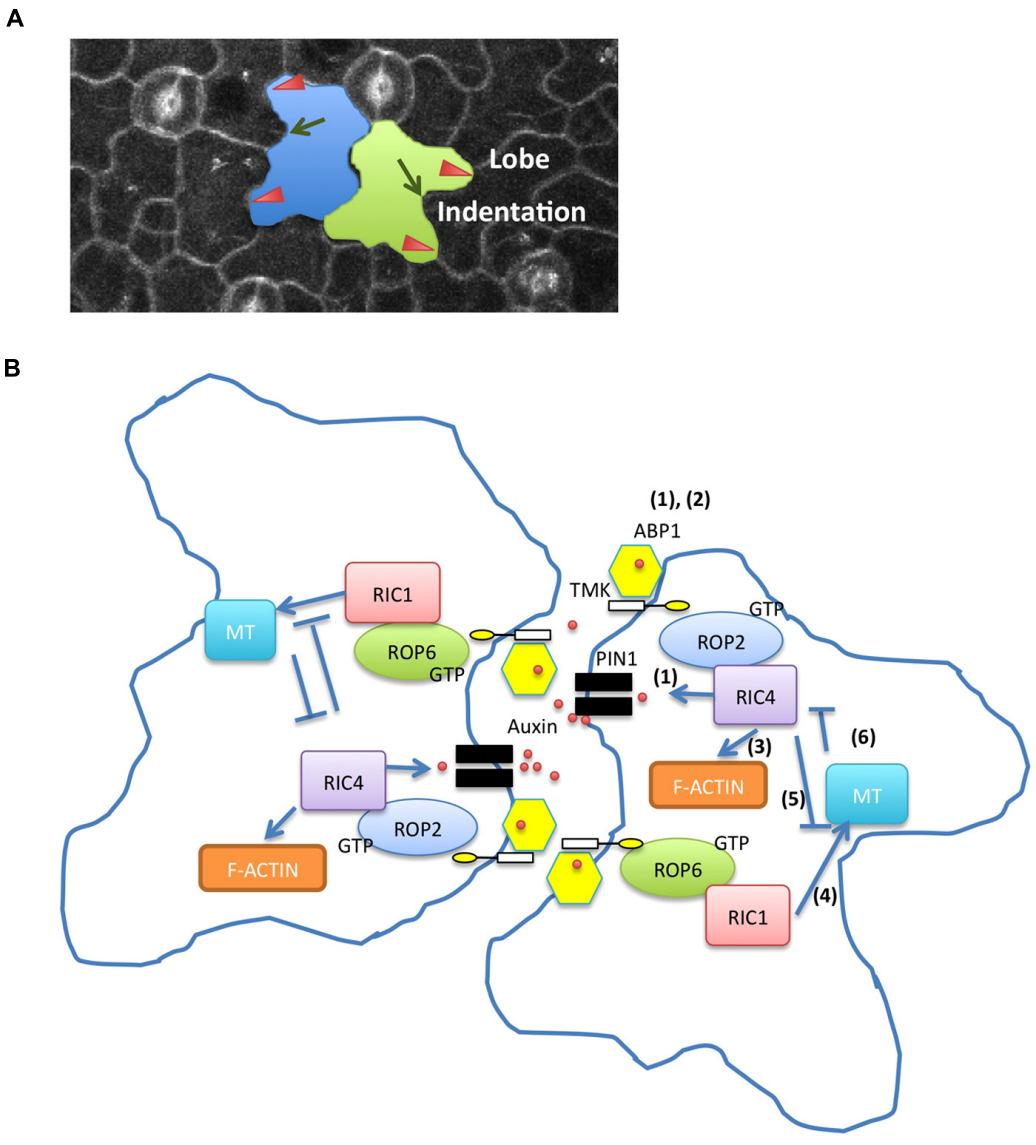
**Two antagonistic Rho-like GTPase from plant (ROP) pathways regulate pavement cell (PC) interdigitation. (A)** Lobes and indentations in *Arabidopsis* PC. Red arrowheads indicate lobes. Green arrows show indentations. **(B)** Auxin activates two antagonistic ROP pathways in PC interdigitation **(1)** Apoplastic auxin localizes to the lobes by PIN1-mediated positive feedback loop. Accumulation of extracellular auxin is generated by the auxin->ROP2->PIN1->auxin feedback loop. **(2)** Auxin controls ROP2 and ROP6 pathways in an ABP1/TMK-dependent manner. **(3)** ROP2 activates RIC4 and promotes assembly of fine cortical actin microfilaments in lobe regions. **(4)** ROP6 activates ROP-interactive CRIB motif containing protein 1 (RIC1) and promotes cortical microtubule (MT) ordering in indenting regions. **(5)** ROP2-mediated pathway inactivates RIC1-mediated MT bundling. **(6)** RIC1-mediated MT organization inhibits ROP2/RIC4 interaction.

**FIGURE 2:**
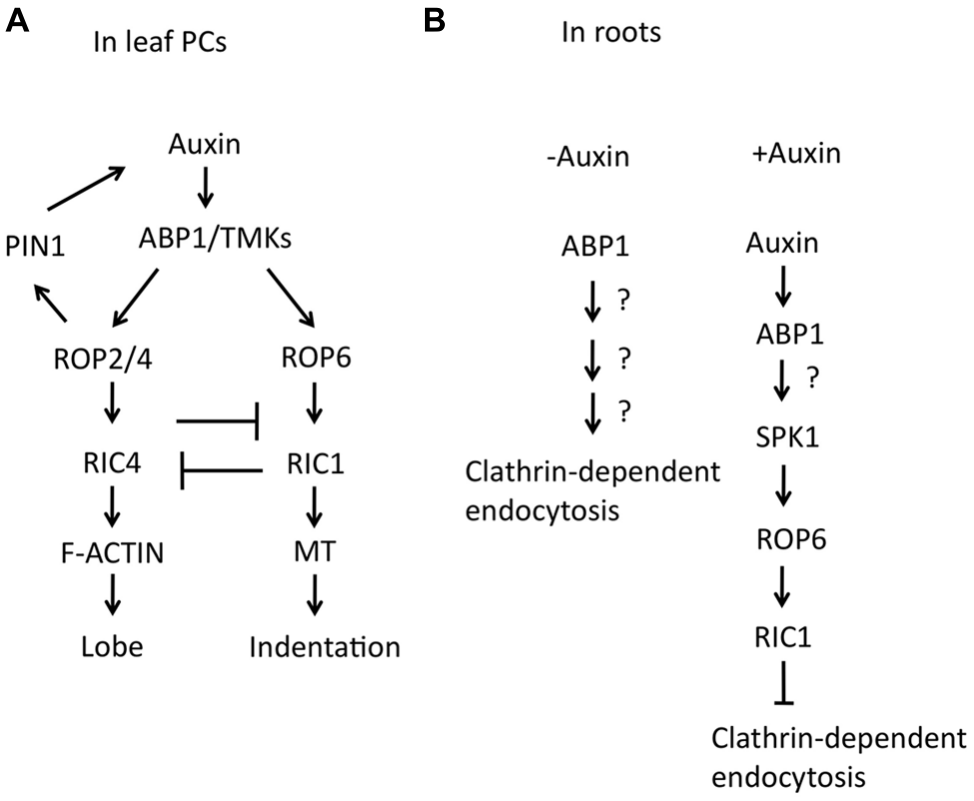
**Roles of auxin for PC interdigitation in leaves and clathrin-dependent endocytosis in roots. (A)** Schematic view for the promoting effects of auxin on two antagonistic ROP pathways and PIN1-mediated positive feedback loop in PC interdigitation. **(B)** Schematic view for an inhibitory effect of auxin on clathrin-dependent endocytosis in roots. Left; In the absence of auxin, ABP1 promotes clathrin-dependent endocytosis. Right; Apoplastic auxin binds to ABP1 and activates SPK1/ROP6/RIC1 pathway, resulting in the inhibition of clathrin-dependent endocytosis.

Leaf epidermal PC is a well-established model system for the study of ROP signaling in *Arabidopsis* (Fu et al., 2002, 2005; Yang, 2008; **Figures 1** and **2A**). These cells form a puzzle piece shape with interdigitated lobes and indentations, and their developmental processes are involved in the establishment of multi-polarity within each cell and the coordination of the multi-polarity between adjacent cells. ROP signaling plays a critical role in regulating the formation of both lobes and indentations during PC patterning. ROP2 and ROP4, two functionally overlapping ROPs, promote the lobe growth by activating the ROP-interactive CRIB motif containing protein 4 (RIC4)-mediated accumulation of fine cortical actin microfilaments (MFs). On the other hand, ROP2 and ROP4 inactivate ROP-interactive CRIB motif containing protein 1 (RIC1)-mediated microtubule (MT) bundling by disrupting its RIC1-association with cortical MTs (Fu et al., 2002, 2005). In contrast, ROP6 promotes cortical MT ordering through RIC1 to restrict radial cell expansion in indenting regions. Both RIC1 overexpression and cortical MT polymerization inhibited ROP2–RIC4 interaction, indicating that RIC1-mediated MT organization antagonized the ROP2/RIC4 pathway (Fu et al., 2005, 2009).

Auxin promotes the interdigitation of PCs in *Arabidopsis* (Xu et al., 2010). Xu et al. (2010) demonstrated that abnormal PC phenotypes of mutants lacking four auxin biosynthetic enzyme genes, *YUCCA*, were suppressed by auxin treatment. They further showed that two antagonistic ROPs, ROP2, and ROP6, are activated in an auxin-dependent manner. The application of exogenous auxin rapidly activates both ROP2 and ROP6 within minutes, suggesting that auxin activates PC interdigitation independent of the well-studied auxin receptors, TRANSPORT INHIBITOR RESPONSE1/AUXIN SIGNALING F-BOX (TIR1/AFBs), which regulate auxin-mediated gene expression in the nucleus. Instead, it was shown that activation of ROP2 and ROP6 by auxin requires AUXIN BINDING PROTEIN1 (ABP1). ABP1 is a 22-kDa glycoprotein, carrying the endoplasmic reticulum (ER) retention signal KDEL sequence and was implicated as an extracellular auxin receptor for the regulation of rapid auxin responses such as auxin promotion of cell expansion (Napier et al., 2002; Braun et al., 2008; Tromas et al., 2009; Robert et al., 2010). A portion of ABP1 is localized to the cell surface, although the majority of it remains in ER (Jones and Herman, 1993; Diekmann et al., 1995; Henderson et al., 1997; Napier et al., 2002). The cell surface action of ABP1 is consistent with a role for ABP1 in the activation of ROPs in PCs. The defects in PC interdigitation in either the *abp1–5*, containing a point mutation (His94->Tyr) in the auxin binding-pocket, or an inducible *ABP1* antisense line were not rescued by auxin, suggesting that ROP2 and ROP6 signaling is regulated downstream of ABP1 (Xu et al., 2010). ROP2 is required for the targeting of the auxin eﬄux transporter, PINFORMED 1 (PIN1), to the lobing regions by inhibiting PIN1 endocytosis, suggesting that the local high levels of auxin, induced by auxin eﬄux, contribute to the promotion of lobe growth (Xu et al., 2010; Nagawa et al., 2012).

Although ABP1 lacks a transmembrane domain, the requirement of ABP1 for the rapid activation of ROP signaling by auxin suggests that ABP1 directly regulates ROP signaling at the PM. It was proposed that apoplastic ABP1 must interact with a transmembrane docking protein to transmit auxin signals to the cytoplasm (Shi and Yang, 2011). A possible docking protein candidate is CBP1, a GPI-anchored protein that binds to the C-terminus of ABP1 and masks the KDEL ER retention signal (Shimomura, 2006). However, CBP1 lacks any motifs to induce downstream signaling, and thus the presence of other proteins that associate with ABP1 or form a protein complex with ABP1 is expected. Recently, (Xu et al., 2014) demonstrated that the transmembrane kinases (TMKs), which belong to the family of receptor like kinase, interact with ABP1 on the cell surface in an auxin-dependent manner to regulate ROP signaling. The four members of the TMK subfamily possess common features; an extracellular domain carrying multiple leucine-rich repeats (LRRs), a transmembrane domain, and an intracellular kinase domain. TMKs have critical roles in cell expansion and cell division downstream of auxin (Dai et al., 2013). The quadruple mutant of *tmk1234* exhibited embryonic lethality with less severity than those phenotypes in *abp1* null mutants. The defects of PC interdigitation of *tmk1234* were not rescued by auxin. Mutations in four TMKs caused a decrease in active ROP2 and ROP6 in the presence of exogenous auxin as well as reduced RIC4 localization at the PM and RIC1 interaction with cortical MTs in PC. These results suggested that TMKs are required for both ROP2 and ROP6 activation in PC patterning. Furthermore, biochemical approaches revealed that the extracellular domain of TMK1 physically interacts with ABP1 in an auxin-dependent manner. Thus, TMK1 is a long-sought docking protein, which is required to transmit the ABP1-mediated auxin signal to the downstream ROP signaling pathways. These findings also confirmed that ABP1 is indeed an extracellular auxin receptor and provide the first example of an extracellular signal that activates ROP signaling at the PM.

The regulation of ROP signaling by extracellular auxin appears to be a common signaling mechanism in plants. Apoplastic ABP1 promotes clathrin-dependent endocytosis, which leads to PIN internalization in roots. Auxin binding to ABP1 inhibits the positive action of ABP1 in endocytosis (Robert et al., 2010; **Figure 2B**). Genetic analysis further revealed that ROP6/RIC1 act downstream of ABP1 to regulate clathrin-dependent endocytosis (Chen et al., 2012). SPIKE1 (SPK1), a ROP guanine nucleotide exchange factor, is required for ROP6 activation by auxin (Lin et al., 2012; **Figure 2B**). The SPK1-ROP6-RIC1 signaling pathway was demonstrated to regulate PIN2 distribution through the regulation of clathrin-dependent endocytosis in roots. It will be interesting to determine whether TMKs are also involved in ROP signaling downstream of auxin that inhibits PIN endocytosis in roots.

Auxin can also regulate root hair elongation through ROP signaling. Root hair derives from a single epidermal hair-forming cell, followed by tip-growth at apex of the cell as observed in pollen tube growth. Immunolocalization of the ROP4 protein and the localization of GFP-ROP2 revealed that these ROPs are localized to the tip of elongating root hair cells. The constitutively-active (CA) form of ROP2, ROP4, and ROP6 induced isotropic growth and elongation of root hair cells, whereas the dominant-negative (DN) form of ROP2 inhibited the tip growth of root hair (Molendijk et al., 2001; Jones et al., 2002; Yang, 2002; Duan et al., 2010) identified the receptor-like kinase FERONIA (FER), as an upstream regulator of ROP signaling by using RopGEF1 as the bait in a yeast two-hybrid screen. Several lines of evidence suggest that the complex formed by FER and RopGEF1 recruits an inactive form of ROP2 and converts it to an active form. Auxin was reported to stimulate root hair elongation (Pitts et al., 1998; Rahman et al., 2002). Root hairs of *fer* mutants are insensitive to exogenous application of auxin, whereas GFP-ROP2 overexpression restored root hair elongation of a *fer* mutant. The *fer* mutants showed decreased accumulation of active ROPs, and the pull-down assay revealed that the inactive (GDP) form of ROP2 preferentially binds to FER. These data suggest that FER is an upstream receptor of ROP signaling in the auxin-dependent root hair development. FER activates NADPH oxidase-dependent production of reactive oxygen species (ROS), which is a second messenger for polar growth (Duan et al., 2010). It would be interesting to investigate whether ABP1-TMKs signaling is involved in the FER-dependent root hair elongation activated by auxin and if so, what the relationship between TMKs and FER is. Recently, Haruta et al. (2014) have reported that a secreted peptide RALF (rapid alkalinization factor) directly interacts with FER to suppress root cell elongation, and RALF acts through FER to inactivate the PM H^+^-ATPase. Auxin promotes H^+^ extrusion through H^+^-ATPase that causes the lower apoplastic pH, followed by the extension of cell wall. Another recent report showed that auxin induces phosphorylation of the H^+^-ATPase as well as hypocotyl elongation in a TIR1/AFB-independent manner (Takahashi et al., 2012). Both auxin-induced cell elongation and H^+^ extrusion depend on K^+^ uptake through inward-rectifying K^+^ channels (Claussen et al., 1997; Philippar et al., 2006). Since it has been reported that the C-terminal region of maize ABP1 modulates K^+^ channels coupling to cytoplasmic pH (pHi; Thiel et al., 1993), ABP1-TMK signaling may control pH through H^+^-ATPase. Thus it will be intriguing to determine whether RALF-FER signaling regulates ROP, and to elucidate the crosstalk between RALF-FER signaling and ABP1-TMK signaling. The molecular mechanism underlying FER-mediated pathway may shed light on how multiple signals are integrated in a specific developmental phase.

### ROP SIGNALING IS REGULATED BY RECEPTOR LIKE KINASES IN POLLEN TUBE GROWTH

*Arabidopsis* ROP1, a pollen-specific member of ROPs, is the central regulator of pollen tube tip growth. The active ROP1 protein is localized in the apical cap of the PM of pollen tubes as an apical cap, corresponding to the expanding region (Lin et al., 1996; Hwang et al., 2005). Inhibition of ROP1 signaling by DN-ROP1 or microinjection of anti-ROP1 caused pollen tube growth arrest, whereas overexpression of ROP1 and CA-ROP1 induced depolarized pollen tube growth (Lin and Yang, 1997; Li et al., 1999; Gu et al., 2003). ROP1 activates two counteracting pathways; the RIC4-dependent F-actin assembly pathway controls the vesicle accumulation required for tip growth, whilst the RIC3-dependent calcium accumulation leads to F-actin disassembly that promotes exocytosis at the tip. (Gu et al., 2005; Lee et al., 2008). Mathematical models and experimental evidence suggest that the RIC4-F-actin pathway contributes to a positive feedback regulation of ROP1 signaling, whereas the RIC3-calcium pathway has a role in a negative feedback regulation of ROP1 signaling (Yan et al., 2009). *REN1* (*ROP1 enhancer 1*) encodes a Rho GTPase-activating protein (RhoGAP), which confines ROP1 activity to the tip of pollen tube. REN1 localizes in vesicles that accumulate in the apex of pollen tubes and at the PM of pollen tube tip, where ROP1 is activated. It was reported that ROP1 function is required for both the localization and the function of REN1, indicating that both spatial distribution and function of REN1 are downstream of ROP1 signaling (Hwang et al., 2008). Thus REN1 appears to participate in the negative feedback regulation of ROP1 signaling. It will be interesting to determine whether REN1 function in the negative feedback is linked to calcium signaling.

Another outstanding question about the regulation of ROP signaling involves the nature of the initial signal that activates ROP1 to promote pollen tube growth. It appears that the signal may also be extracellular, as another subfamily of RLK has been implicated in the activation of ROP1 signaling in pollen tubes. The two pollen-specific RLKs, LePRK1 and LePRK2 (for *Lycopersicon esculentum* pollen receptor kinase 1 and 2) were discovered (Muschietti et al., 1998). LePRK1 and LePRK2 are both localized at the surface of elongating pollen tubes. LePRK1 and LePRK2 are coimmunoprecipitated in pollen that suggesting that these two receptors interact with each other (Wengier et al., 2003). The down-regulation of *LePRK2* by antisense of *LePRK2* expression caused reduced pollen germination and defects in pollen tube growth (Zhang et al., 2008). A kinase partner protein (LeKPP), the pollen specific homolog of RopGEF from tomato, associates with the cytoplasmic region of LePRK2 in pollen. Pollen specific promoter-driven *LeKPP* resulted in depolarization of pollen tube with abnormal actin assembly (Kaothien et al., 2005). AtRopGEF12, an *Arabidopsis* homolog of LeKPP, interacts with an *Arabidopsis* pollen receptor kinase AtPRK2a. The C-terminally truncated form of AtRopGEF12 disturbed pollen tube morphology, whereas full-length AtRopGEF12 caused slightly wider pollen tubes, indicating that the C terminus has an inhibitory function for GEF activity. A phospho-mimicking mutation at a conserved serine residue in the C terminus of AtRopGEF12 caused the loss of inhibitory effects. Coexpression of AtRopGEF12 and AtPRK2a resulted in isotropic growth of pollen tubes as seen in CA-ROP overepressing lines. Since coexpression of AtPRK2a and an N-terminally truncated form of AtRopGEF12 did not cause the isotropic pollen tube growth, the N terminal region of AtRopGEF12 appears to play a positive role for the activation of ROP signaling in the pollen tube growth (Zhang and McCormick, 2007). These data suggest that AtPRK2a binds to the C-terminal region of AtRopGEF12, and the self-inhibitory function of GEF activity is lost by phosphorylation at an invariant serine residue, and then the N terminal region of AtRopGEF12 activates downstream ROP signaling. AtPRK2a possesses a kinase activity (Chang et al., 2013). A recent report showed that not only the C-terminal region but also the juxtamembrane (JM) domain of AtPRK2a is critical for its interaction with AtRopGEF12 and the subcellular distribution of AtPRK2a at the PM, suggesting that AtPRK2a can interact with AtRopGEF12 without phosphorylation (Zhao et al., 2013).

There are fourteen members of the RopGEFs family in *Arabidopsis*. Transient expression of five AtRopGEFs causes swollen phenotypes in tobacco pollen, as seen in ROP1- or CA-ROP1-overexpressed pollen (Lin and Yang, 1997; Li et al., 1999; Gu et al., 2003). AtRopGEF1 overexpression induced the severe swollen phenotypes in a ROP1-dependent manner. The PRONE domain of AtRopGEF1 exhibited GEF activity on ROP1. These data suggest that RopGEF1 directly activates ROP1 (Gu et al., 2006). Chang et al. (2013) showed that AtPRK2 (also referred to as AtPRK2a) induces ROP1 activity and pull-down assay revealed that AtPRK2 physically interacts with AtRopGEF1 and ROP1. AtPRK2 directly phosphorylates AtRopGEF1 in the C-terminal region, which is critical for AtRopGEF1 function (Gu et al., 2006; Chang et al., 2013). Furthermore, CA-AtRopGEF1 (PRONE domain of AtRopGEF1) rescued pollen germination defects in the DN-AtPRK2 (a kinase domain-deleted form of AtPRK2) overexpressing line (Chang et al., 2013). These data suggest that the AtPRK2-AtRopGEF1-ROP1 signaling pathway controls pollen tube growth.

A fascinating outstanding question remains the nature of the extracellular signal that regulates RLKs in pollen tube growth. LAT52, a cysteine-rich extracellular protein from pollen, interacts with LePRK2 (Muschietti et al., 1994; Tang et al., 2002). LeSHY, a leucine-rich repeat protein from pollen, and LeSTIG1, a small cysteine-rich protein from pistil, can bind the extracellular domains of LePRK1 and LePRK2, and exogenous application of LeSTIG1 promotes pollen tube growth (Tang et al., 2004; Huang et al., 2014). It remains unknown whether these signals regulate ROP signaling in tomato. It may be intriguing to identify the signal that binds to AtPRK2 and to examine whether the signal regulates AtPRK2-AtRopGEF1/AtRopGEF12-ROP1 pathway in *Arabidopsis* pollen tube growth.

### PAN1 RLK AND ROP SIGNALING REGULATE ASYMMETRIC CELL DIVISION IN MAIZE

The development of stomatal complex in maize (*Zea mays*) is tightly associated with the coordination of asymmetric cell division. Stomatal complexes consist of a pair of guard cells flanked by a pair of subsidiary cells that regulate the function of guard cells. At first, guard mother cell (GMC), which eventually produces the guard cell pair, is formed by asymmetric cell division. Before formation of a guard cell pair, the nuclei of subsidiary mother cells (SMCs), which are lateral neighbors of GMC, are polarized toward GMC as well as forming a cortical F-actin patch at the boundaries between GMC and SMCs. And then the subsequent asymmetric cell division produces the subsidiary cells.

PANGLOSS1 (PAN1), a leucine-rich repeat receptor-like protein with inactive kinase domain, is localized in SMCs and newly formed subsidiary cells at sites of contact with GMCs. PAN1 and actin patches appeared after GMC formation and before nuclear polarization, and PAN1 patches were detectable before actin patches. Thus, PAN1 promotes the premitotic polarization of SMCs by receiving the cue from the GMC rather than intrinsic cues (Cartwright et al., 2009). Given the roles of ROPs in cell polarization and their association with RLKs, ROP signaling was expected to contribute to the polarization of SMCs. Nine ROPs were identified in maize. Two closely related ROPs, ROP2, and ROP9, play the roles in promoting the polarization of SMCs with PAN1. Although mutations in *ROP2* or *ROP9* alone caused no significant defects in the subsidiary cells, the double mutants *rop2/rop2rop9/+* exhibited a slightly higher frequency of abnormal subsidiary cells. These mutations significantly enhanced the *pan1* phenotype of SMC division. In SMCs, ROP proteins localize at sites of contact with GMC, which is similar to the PAN1 localization. ROP patches are formed after the formation of PAN1 patches. The accumulation of ROP in SMCs was altered in *pan1* mutant, whereas the localization of PAN1 was not disturbed in *rop2/rop2rop9/+* mutants. Furthermore, biochemical approaches revealed that PAN1 and ROPs are physically associated in maize leaf extracts (Humphries et al., 2011). These data suggest that ROP2 and ROP9 function downstream of PAN1 to promote the premitotic polarization of SMCs and the activity of these ROPs can be spatially regulated. PAN1 lacks a functional kinase domain, and thus is expected to act with other proteins in signal transduction, but currently there is no knowledge of signaling protein(s) that form a complex with PAN1. Upstream signaling molecules that regulate the PAN1-ROP2/ROP9 pathway are unknown. Recently, auxin signaling is shown to be involved in stomatal patterning in *Arabidopsis* (Le et al., 2014). Since the polar localization of maize PIN1 (ZmPIN1a) in subsidiary cells has been reported (Sutimantanapi et al., 2014), it is tempting to speculate that auxin might be an upstream molecule for PAN1-ROP2/ROP9 pathway, similar to the TMK-ROP pathways in *Arabidopsis*.

### ROPs NEGATIVELY REGULATE ABSCISIC ACID SIGNALING

The phytohormone ABA is a pivotal hormone that is involved in seed germination, plant development, and abiotic stress responses. Several putative ABA receptors including those localized to the PM have been proposed, and biochemical studies have also implied the existence of both cell surface and intracellular ABA receptors. However, only the pyrabactin resistance1 (PYR)/PYR1-like (PYL)/regulatory component of ABA receptor (RCAR) family ABA receptors, which are localized to the cytoplasm or nucleus, have been widely accepted (Fujii et al., 2009; Ma et al., 2009; Nishimura et al., 2009; Park et al., 2009). The ABA-mediated interaction of these receptors with A-type protein phosphatase 2Cs (PP2Cs) results in the inhibition of PP2Cs, which are negative regulators of ABA signaling. The SNF1-related protein kinase 2 (SnRK2)-type protein kinases can then activate downstream signaling.

Evidence suggests that ROP GTPases also participate in the regulation of ABA signaling mediated by the PYR/PYL/RCAR family ABA receptors. Expression of a dominant-positive mutant of ROP6/AtRac1 inhibited ABA-mediated stomatal closure and cytoskeletal reorganization, whereas expression of a DNform of ROP6 caused ABA response in an ABA-independent manner, indicating that ROP6 signaling negatively regulates ABA signaling (Lemichez et al., 2001). *ROP10* loss of function mutants exhibit hypersensitivity to ABA in root elongation response, and stomatal closure. These mutants also displayed the enhanced seed dormancy, which is regulated by ABA and enhanced responses to ABA inhibition of seed germination. CA-ROP10 and DN-ROP10 mutants exhibited the insensitivity and hypersensitivity to ABA, respectively. The promoter-GUS analysis revealed that the *ROP10* promoter activity in root tips is down-regulated by ABA (Zheng et al., 2002). These results suggest that a mutual inhibition between ROP10 signaling and ABA signaling. The ROP10-mediated pathway negatively regulates ABA signaling, while *ROP10* transcripts are down-regulated by ABA. ROP11, which belongs to the same subfamily of ROP10, has been shown to be another negative regulator of ABA signaling in multiple ABA responses. The loss of function mutants of *ROP11* exhibited hypersensitivity to ABA similarly to *ROP10* mutants. Several lines of evidence show that ROP11 and CA-ROP11 directly interact with ABI1 and ABI2, which are members of the PP2Cs family (Li et al., 2012a,b; Yu et al., 2012). *In vitro* phosphatase activity assay showed that inhibition of ABI1 by RCAR1 was alleviated by CA-ROP11, and ROP11 enhanced the ABI2 activity (Li et al., 2012b; Yu et al., 2012). RopGEF1 and RopGEF4 have been reported to be upstream regulators of ROP11 in ABA-mediated stomatal closure (Li and Liu, 2012). These data suggest that ROP signaling protects the hub of ABA signaling. Furthermore, mutations in *FER* caused hypersensitivity to ABA in root elongation responses and stomatal closure, as well as higher level of ROS accumulation in guard cells (Yu et al., 2012). Since RopGEF1 has been reported to interact with FER (Duan et al., 2010), FER-mediated signaling may regulate ABA responses through ROPs. Although ABA itself may be a regulator of ROP signaling in a negative feedback loop, the signals that activate specific ROPs in ABA signaling remain unknown. Given the occurrence of FER-RopGEF-ROP signaling at the PM, these results raise the possibility that ROPs may regulate ABA signaling at the PM too.

## CONCLUDING REMARKS

Rho-like GTPase from plants signaling acts downstream of diverse signaling pathways such as auxin, ABA, and cytosolic calcium. Increasing evidence reveals that several ROPs are involved in RLK-mediated signaling. Given the diverse roles of ROP signaling in cellular processes such as actin accumulation, ordering of MTs, calcium concentration, H_2_O_2_ production, gene expression, all of which lead to symmetry breaking (Yang, 2002; Yang and Lavagi, 2012), multiple environmental and developmental signals that perceived by RLKs may be regulated by RLK-ROP modules. One of aspect yet to be examined remains the establishment of RLK-ROP modules as common features in plants. Thus far, FER, TMK, and AtPRK2a in *Arabidopsis*, LePRK1 and LePRK2 in tomato, and PAN1 in maize have been shown to be involved in ROP signaling, suggesting that the framework of RLK (or receptor like protein)-ROP is conserved among higher plants for acquisition of cell polarity (**Table 1**). The identification of RLKs or receptor-like proteins that interact with upstream regulators of ROPs like RopGEFs will be critical to further our understanding of ROP-mediated signaling. Another critical facet of RLK-ROP modules regards the roles of the interaction between RLKs and upstream regulators of ROP. Binding of the C terminal region of AtRopGEF12 with AtPRK2a results in the loss of C terminal inhibition of AtRopGEF12 activity and contributes to the activation of downstream ROP signaling in the tip growth of pollen tube (Zhang and McCormick, 2007; Chang et al., 2013). In root hair development, the inactive form of ROP2 preferentially binds to the complex of FER and AtRopGEF1, as compared with the active form of ROP2. The conversion of GDP-bound to GTP-bound ROP2 may be facilitated in an upstream signal-dependent manner, and then the activated ROP2 may be released for downstream pathway (Duan et al., 2010). In PC interdigitation, auxin binding to ABP1 is shown to be required for the formation of the ABP1–TMK complex, as well as for the subsequent activation of ROP signaling (Xu et al., 2014). Understanding the complexity of RLK-ROP pathways will provide new insights into how a variety of signals can regulate ROP signaling.

**Table 1:** A list of known and putative RLK–RopGEF–ROP combinations and their roles in various plant development.

Signal	Receptor	RopGEF	ROP	Function	Plants	Reference
Auxin	ABP1/TMK	Unknown	ROP2, ROP4, ROP6	PC interdigitation	*Arabidopsis thaliana*	Xu et al. (2010, 2014)
Auxin?	FER	RopGEF 1	ROP2	Root hair development	*Arabidopsis thaliana*	Duan et al. (2010)
LAT52?, LeSTIG1, LeSHY	LePRK1/LePRK2	LeKPP	Unknown	Pollen tube elongation	*Lycopersicon esculentum*	Muschietti et al. (1994, 1998), Tang et al. (2002, 2004), Kaothien et al. (2005)
Unknown	AtPRK2a	RopGEF 1 RopGEF 12	ROP1	Pollen tube elongation	*Arabidopsis thaliana*	Gu et al. (2006), Zhang and McCormick (2007), Chang et al. (2013)
Unknown	PAN1	Unknown	ROP2, ROP9	Stomatal complex development	*Zea mays*	Cartwright et al. (2009), Humphries et al. (2011)
Unknown	FER	RopGEF 1	ROP 10, ROP 11	ABA signaling	*Arabidopsis thaliana*	Zheng et al. (2002), Li and Liu (2012), Yu et al. (2012), Li et al. (2012a,b)

## Conflict of Interest Statement

The authors declare that the research was conducted in the absence of any commercial or financial relationships that could be construed as a potential conflict of interest.
